# An acetonic extract and secondary metabolites from the endolichenic fungus *Nemania* sp. EL006872 exhibit immune checkpoint inhibitory activity in lung cancer cell

**DOI:** 10.3389/fphar.2022.986946

**Published:** 2022-09-08

**Authors:** Mücahit Varlı, Huong T. Pham, Seong-Min Kim, İsa Taş, Chathurika D. B. Gamage, Rui Zhou, Sultan Pulat, So-Yeon Park, Nüzhet Cenk Sesal, Jae-Seoun Hur, Kyo Bin Kang, Hangun Kim

**Affiliations:** ^1^ College of Pharmacy, Sunchon National University, Sunchon, South Korea; ^2^ College of Pharmacy, Sookmyung Women’s University, Seoul, South Korea; ^3^ Faculty of Arts and Sciences, Department of Biology, Marmara University, Istanbul, Turkey; ^4^ Korean Lichen Research Institute, Sunchon National University, Sunchon, South Korea

**Keywords:** *Nemania* sp., benzo[a]pyrene, programmed death-ligand 1, inducible T Cell costimulator ligand, radianspenes C and D, dahliane D, immune checkpoints

## Abstract

**Background:** Endolichenic fungi (ELF), which live the inside the lichen thallus, contain many secondary metabolites that show various biological activities. Recent studies show that lichen and ELF secondary metabolites have antioxidant, antibacterial, antifungal, cytotoxic, and anticancer activities.

**Purpose:** Here, the effects of an ELF extract and its bioactive compounds were investigated on the H1975 cell line focusing on immune checkpoint marker inhibition.

**Methods:** An ELF was isolated from the host lichen *Bryoria fuscescens* (Gyelnik) Brodo and D. Hawksw and identified the species as *Nemania* sp. EL006872. The fungus was cultured on agar medium and acetonic extracts were obtained. Secondary metabolites radianspenes C and D, and dahliane D, were isolated from the crude extract. The biological effects of both the crude extract and the isolated secondary metabolites were evaluated in cell viability, qRT-PCR assays, flow cytometry analysis and western blotting.

**Results:** The cell viability assay revealed that extracts from *Nemania* sp. EL006872 and the isolated secondary compounds had low cytotoxicity. The crude extract, radianspenes C and D, and dahliane D, suppressed expression of mRNA encoding PD-L1 and aromatic hydrocarbon receptor (AhR), and surface expression of PD-L1 protein by cells exposed to benzo[a] pyrene. Radianspenes C and D, and dahliane D, reduced expression of AhR, PD-L1, ICOSL, and GITRL proteins by H1975 lung cancer cells, as well as exerting anti-proliferative effects.

**Conclusion:** Radianspenes C and D, and dahliane D, bioactive compounds isolated from *Nemania* sp. EL006872 ELF, have the potential for use as immunotherapy and immunoncology treatments.

## Introduction

Exposure to environmental pollutants and tobacco smoke is an important public health problem involving major pollutant benzo[a]pyrene (BaP) (Poirier, 2004; Taş et al., 2019b). Sources of BaP include coal tar (Zieliński et al., 1996), cigarette smoke, automobile exhaust fumes (Tancell et al., 1995), smoke generated by combustion of organic material, and grilled food (Aygün and Kabadayi, 2005). Exposure to BaP causes lung cancer and systemic inflammation (Shi et al., 2017). Lung cancer is the most common cause of cancer-related death worldwide (Gomes et al., 2014). BaP-mediated modulation of gene transcription occurs through activation of the aryl hydrocarbon receptor (AhR) or via DNA damage (Shimizu et al., 2000; Hockley et al., 2007; Vázquez-Gómez et al., 2018). Upon exposure to BaP and tobacco smoke, AhR induces expression of an important immune checkpoint, programmed death ligand 1 (PD-L1) (Wang et al., 2019). Therefore, development of new effective agents or cancer immune therapy treatments is required (Jain et al., 2018).

Lichens, symbiotic organisms comprising fungi and algae (or cyanobacteria), have attracted much attention as a source of therapeutic agents for the treatment of numerous diseases, including cancer (Zhou et al., 2017; Yang et al., 2018b, 2018a; Taş et al., 2019a; Tas et al., 2019; Lee et al., 2021). Similar to endophytes living inside plants, endolichenic fungi (ELF) live inside lichen thalli (Honegger et al., 2013). Many studies have identified secondary metabolites in ELF (Wijeratne et al., 2012, 2010; Wang et al., 2010; Li et al., 2015, 2012; He et al., 2012; Jiao et al., 2015). In recent years, it has been reported that microbes as produce different secondary metabolites, including antioxidant, antiviral, anti-Alzheimer, anti-inflammatory, UV protectant, antimicrobial and anticancer activities (Padhi et al., 2019a; Agrawal et al., 2020). Endolichenic fungi secondary metabolites can be classified into the different chemical groups, steroids, terpenoids, xanthones, quinones, peptides, sulphur-containing chromenones, etc (Yang et al., 2018b; Padhi et al., 2019b). More than 40,000 terpenoid compounds have been isolated from natural sources and, especially, diterpenoids can be found in all areas of life, and are secondary metabolites of fungi and plants (Zhang and Feng, 2022). Due to their structural diversity and bioactivity, diterpenoids may be potential alternative therapies. Studies show that fungal diterpenoids have cytotoxic, anti-inflammatory, antimicrobial, anti-MRSA, antiviral, and antihypertensive properties (Ling et al., 2014; Büschleb et al., 2016; Hu et al., 2020; Min et al., 2020).

Cancer immunotherapy aims to strengthen the human immune system, thereby enabling it to eliminate cancer cells (Li et al., 2016). The fundamental rationale underlying cancer immunotherapy lies in utilization of specific antibodies and T cells that enable the immune system to discriminate the tiniest biochemical differences between cancer and normal cells (Rojas-Martínez et al., 2017). Tumors use multiple immune regulatory mechanisms to inhibit their antitumor immune effects. Immune checkpoints are inhibitory pathways that physiologically balance co-stimulatory pathways to fine-tune immune responses. Regarding the two components of an immune checkpoint, molecules expressed by immune cells are often referred to as immune checkpoint receptors, while those expressed by antigen-presenting cells, tumor cells or other cell types are called immune checkpoint ligands (Foy et al., 2016; Gibney et al., 2016). Overexpression of immune checkpoint ligands by tumor cells affects tumor-specific T cell immunity in the cancer microenvironment. Since most tumor immune escape mechanisms based on these checkpoints block effector cell functions, antitumor immunity can be restored by antibodies that block inhibitory receptor-ligand interactions, thereby inactivating the immune checkpoints (Carosella et al., 2015).

In this study, the aim was to identify the immunotherapeutic potential of *Nemania* sp*.* EL006872 extracts, and its diterpenoids metabolites, radianspenes C and D, and dahliane D, particuraly in the context of immune checkpoint inhibition. To the best of our knowledge, few studies have examined the efficacy of radianspenes and dahlianes as potential immunotherapy agents.

## Materials and methods

### Sample collection, isolation and identification of the endolichenic fungus

The lichen samples of *Bryoria fuscescens* (Gyelnik) Brodo and D. Hawksw were collected in 2019 during the field trips in Mt. Uludağ (40˚06’00.73″N, 29˚16’59.79″E), Bursa, Turkey, organized by Prof. Sesal from Dept. of Plant Diseases and Microbiology, Marmara University, Istanbul, Turkey. Voucher samples (KoLRI 052660) has been deposited at the Korean Lichen and Allied Bioresource Center (KOLABIC) in the Korean Lichen Research Institute (KoLRI), Sunchon National University, Korea (https://cc.aris.re.kr/kolabic). Endolichenic fungi were isolated with the surface sterilization method (Guo et al., 2003).

Fungus internal transcribed spacer sequencing conducted as already depicted (Yang et al., 2018b). The endolichenic fungus is cultured on potato dextrose agar (PDA) (BD Difco, Sparks, MD, United States). DNA was extracted from ELFs using a DNeasy Plant Mini Kit following the manufacturer’s protocols (Qiagen, Hilden, Germany). The ITS (internal transcribed spacer) region of the rDNA was amplified with the common using primers ITS1F (5′-CTT​GGT​CAT​TTA​GAG​GAA​GTA​A-3′) (Gardes and Bruns, 1993) and LR5 (5′-ATC​CTG​AGG​GAA​ACT​TC-3′) (Vilgalys and Hester, 1990). The strain used in this study (EL006872), was identified as *Nemania* sp. by the BLAST search of the ITS sequence, which showed 99.81% similarity against *Nemania* sp. genotype 184 (GenBank Accession No. JQ761782.1). The ITS sequence of *Nemania* sp. EL006872 is available in Supplementary Table S1.

### Pure culture and metabolites extraction

Potato dextrose agar (PDA) (BD Difco, Sparks, MD, United States) powder (39 g) was prepared into 1 L distilled water, which was then boiled and mixed to dissolve the powder. The mixture was autoclaved. The autoclaved PDA solution was cooled down to 55–60°C and then directly transferred into the petri dishes. Petri dishes were cooled to room temperature. ELFs were transferred to be cultured on agar in each petri dish. The dishes were incubated at 25°C for 3–4 weeks. ELF mycelia were grown on PDA and then frozen with agar and lyophilized. The lyophilization was separated into water. Then, acetone was added to samples tubes, and the tubes were shaken for around 2 h. Each sample was then filtered to separate from solid particles. Crude extracts of ELFs were then obtained by rotary evaporator. Finally, the crude extracts were dissolved in 100% DMSO for use.

### Isolation and structure elucidation

The dried crude extract (2.7 g) was suspended in water, then successively extracted with *n-*hexane and ethyl acetate (EtOAc). A Waters 600 HPLC system (Waters Co., Milford, MA, United States) equipped with a Hector-M C_18_ column (250 mm × 10 mm, 5 μM, RS Tech, Daejeon, Korea) or a Spursil C_18_ EP column (250 mm × 10.0 mm, 5 μM, Dikma Technologies, Foothill Ranch, CA, United States) was used for the purification of compounds **1**–**3** from the EtOAc fraction. The EtOAc fraction (78.9 mg) was further separated by preparative HPLC (Hector C_18_ column, 4 ml/min, MeCN-0.1% formic acid in H_2_O 20:80 → 80:20) into five subfractions (E1– E5). Compounds **1** (1.0 mg, t_R_ = 10.0 min) and **2** (1.3 mg, t_R_ = 8.0 min) were purified from the subtraction E4 by preparative HPLC (Spursil C1_8_ EP column, 4 ml/min, MeCN-0.1% formic acid in H_2_O 45:55 → 58:42). Compound **3** (0.7 mg, t_R_ = 11.5 min) was obtained from the subfraction E4 by preparative HPLC (Spursil C1_8_ EP column, 4 ml/min, MeCN-0.1% formic acid in H_2_O 45:55 → 58:42). NMR spectra were obtained using a Bruker Avance III HD 500 spectrometer (Bruker BioSpin, Billerica, MA, United States) (Supplementary Table S2; Supplementary Figures S1-S13).

### Cell culture

H1975 non-small lung cancer cell line was used in this research. H1975 cell was cultured in RPMI (GenDepot, Katy, TX, United States) supplemented with 10% fetal bovine serum (GenDepot, Katy, TX, United States) and 1% penicillin streptomycin solution. Cells were incubated in 5% CO_2_ in a humidified atmosphere at 37°C.

### Cell viability assay

Extract and pure compounds were dissolved in DMSO (Sigma-Aldrich, St. Louis, MO, United States). Cells were seeded at a density of 3 x 10^3^ cells/well in 96-well plates, grown overnight, and then treated with crude extract and pure compounds. After incubation, 15 μL methyl thiazolyl tetrazolium (MTT) reagent (Sigma-Aldrich) was added to each well, and the samples were incubated for an additional 4 h. After 4 h, the supernatants were removed, and formazan crystals were dissolved in 150 µL DMSO. Absorbance was measured at 570 nm and was determined using a microplate reader with Gen5 (v2.03.1) software (BioTek, Winooski, VT, United States).

### Clonogenic assay

H1975 cells were seeded at a density of 1000–1500 cells/well in RPMI 1640 and incubated overnight. After 72 h, media containing the crude extract and indicated compounds or DMSO was replaced with fresh medium for 12 days. Colonies were fixed with methanol, and stained with 0.5% crystal violet. Then, the plating efficiency and survival rate of the control group and treatment group cells were determined. Next, the plating efficiency of the control group and treatment groups were determined (Le et al., 2022).

### Quantitative reverse-transcription PCR

Quantitative RT-PCR (qRT-PCR) was conducted as already depicted. H1975 were plated at a density of 2 x 10^5^ cells/well on 6-well plate and grown overnight. Cells were treated with crude extract, pure compounds, and benzo[a]pyrene or DMSO. Total RNA was isolated from H1975 cells using RNAiso Plus (TaKaRa, Otsu, Japan). 1 mg of RNA was converted to cDNA using M-MLV reverse transcriptase (Invitrogen, Carlsbad, CA, United States). qPCR was performed using SYBR Green (Enzynomics, Seoul, Korea). qRT-PCR reactions and analyses were performed on a CFX instrument (Bio-Rad, Hercules, CA, United States). Primers used for qRT-PCR were as follows: GAPDH (forward) 5′-atc​acc​atc​ttc​cag​gag​cga-3′ and (reverse) 5′-agt​tgt​cat​gga​tga​cct​tgg​c-3`; PD-L1 (forward) 5`-gga​gat​tag​atc​ctg​agg​aaa​acc​a-3` and (reverse) 5`-aac​gga​aga​tga​atg​tca​gtg​cta-3`; AhR (forward) 5`-att​gtg​ccg​agt​ccc​ata​tc-3` and (reverse) 5`-aag​cag​gcg​tgc​att​aga​ct-3`.

### Surface protein expression

H1975 were plated at a density of 2 × 10^5^ cells/well on a 6-well plate and grown overnight. Then, cells were treated with crude extract, radianspenes C and D, and dahliane D 4 h prior to exposure benzo[a]pyrene (1 µM) and then cells were incubated for 72 h. H1975 cell line was incubated for 72 h and collected in PBS by trypsin digestion. After washing three times, cell pellets were resuspended in 2% BSA and 100 μl aliquot (1 cells  ×  10^6^ cells) was used for antibody labeling with anti-PD-L1 antibody (Cell signaling, #13684) at 4 °C for 30 min in the dark. After washing with FACS buffer twice and cell was incubated with secondary antibody anti-Rabbit IgG (H + L) (Cell signaling, Alexa flour 488 conjugate, #4412) at 4 °C for 30 min in the dark. After washing steps, the labeled cells were analyzed by flow cytometry using Cytoflex flow cytometer (Beckman Coulter Life Sciences, Indianapolis, IN, United States) and CytExpert 2.0.0.152 software.

### Western blotting

Cells were cultured in a 6-well plate for 12 h, treated with 7.5, 15, and 30 µM concentrations of radianspenes C and D, and dahliane D for 72 h, washed twice with cold PBS, and lysed in lysis buffer. Primary antibodies (PD-L1 (Cell signaling, #13684), ICOSL (Sigma-Aldrich, WH0023308M1), GITRL (Abcam, ab25948), AhR (Cell signaling, #83200), and GAPDH (Cell signaling, #5174)) against were used to probe membranes. After membranes incubated with secondary antibodies (Thermo Fisher Scientific) and detected with using an Immobilon Western Chemiluminescent HRP Substrate Kit (Merck Millipore, Germany), and luminescence imaging (iBright™ FL1000 Imaging System, Thermo Fisher Sciences). Multi-Gauge 3.0 was used to assess band densities, which were then normalized to GAPDH levels in each sample. The values are given in arbitrary densitometric units that correlate to the signal intensities.

## Results

### *Nemania* sp. EL006872 crude extract shows immune checkpoint inhibitory activity

**FIGURE 1:**
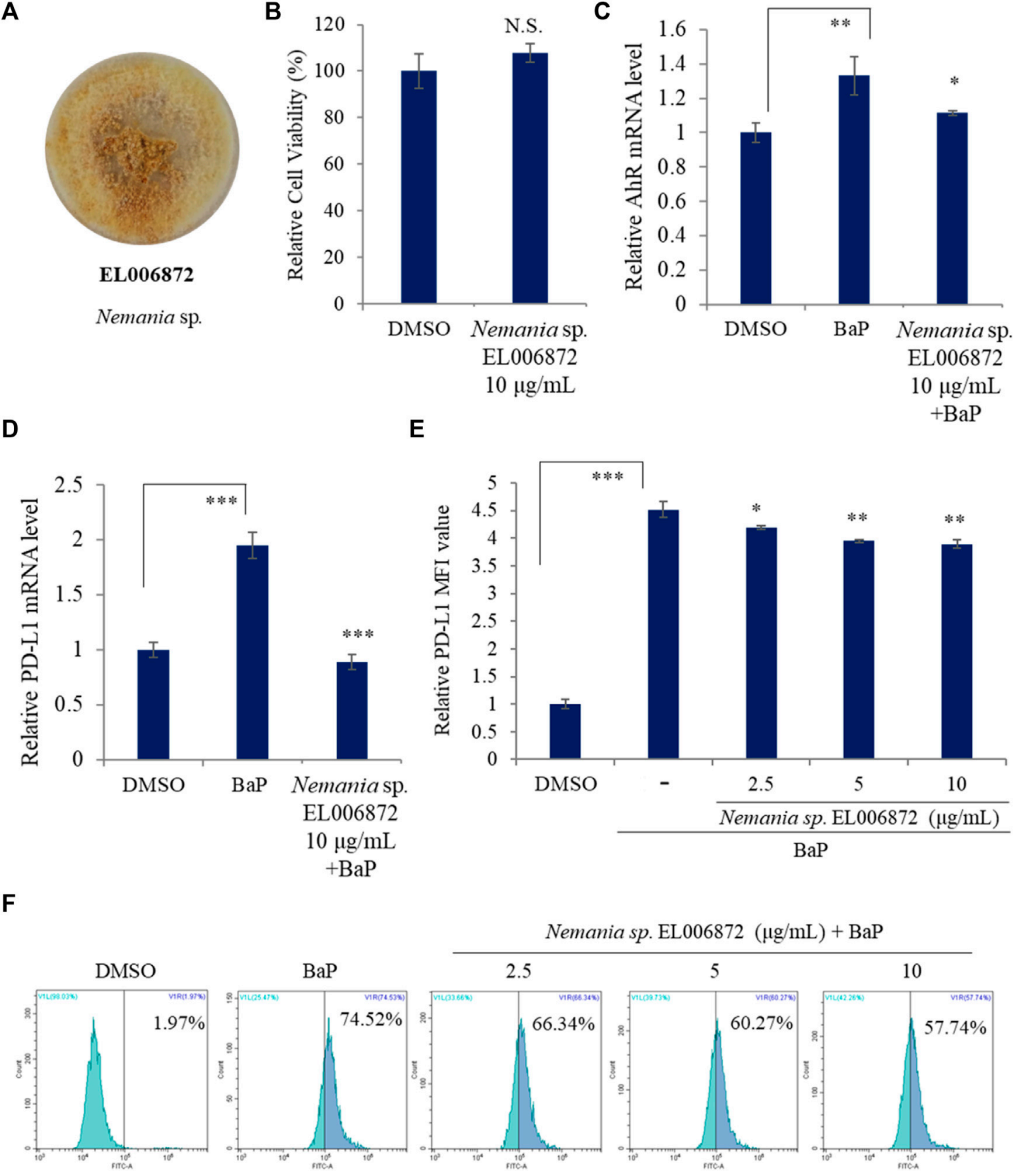
*Nemania* sp. EL006872 inhibits expression of PD-L1 and AhR. **(A)** Images of the isolated endolichenic fungus EL006872. **(B)** Effect of *Nemania* sp. EL006872 crude extract on the viability of H1975 cells. **(C,D)** Effect of *Nemania* sp. EL006872 crude extract on PD-L1 and aromatic hydrocarbon receptor (AhR) mRNA in benzo[a]pyrene (BaP) exposed H1975 cells. **(E,F)** Effects of *Nemania* sp. EL006872 crude extract on surface protein level of PD-L1 in BaP exposed H1975 cells. MFI: mean fluorescence intensity. Data are presented as the mean ± S.D. (standard deviation); *n* = 3. *p < 0.05; **p < 0.01; ***p < 0.001; NS, no significant difference compared with the BaP-treated group or between indicated group.

Figure 1A shows the *Nemania* sp. EL006872 culture. First of all, cell viability assay using acetonic extract of *Nemania* sp. EL006872 was performed to determine the cytotoxic activity of the extract (10 μg/mL). As a result, acetonic extracts of the *Nemania* sp. EL006872 that maintained H1975 cell viability were tested for future experiments (Figure 1B).

Exposure to benzo[a]pyrene (BaP) increases expression of PD-L1 by lung epithelial cells, likely mediated via AhR (Wang et al., 2019). Recent reports suggest that aromatic hydrocarbon receptor (AhR) interacts with metabolites and derivatives such as kynurenine (Esser, 2016; Kawajiri and Fujii-Kuriyama, 2017; Gutiérrez-Vázquez and Quintana, 2018). AhR induces expression by BaP. Overexpression of AhR in many cancers triggers production of toxic metabolites via overexpression of CYP1A1 and CYP1B1 (Harrigan et al., 2004). H1975 non-small lung cancer cell line was used because previous studies show that it overexpresses PD-L1 (Chen et al., 2015; Yan et al., 2016). A hall mark of cancer is that the cells can evade attack by host immune cells. Carcinoma cells may express many immune inhibitory signaling pathways that induce immune cell apoptosis and dysfunction. Programmed death ligand-1 (PD-L1) binds to programmed death-1 (PD-1) expressed by dendritic cells, B cells, T cells, and the natural killer T cells to inhibit their anticancer effects (Chen et al., 2016).

Next, the effects of *Nemania* sp. EL006872 crude extract on expression of AhR and PD-L1 mRNA in BaP exposed H1975 cells were examined. qRT-PCR revealed that expression of PD-L1 and AhR mRNA in BaP exposed H1975 cell was downregulated by pretreatment of acetone extracts of *Nemania* sp. EL006872 (Figures 1C,D). Subsequently, in order to observe the surface protein expression, flow cytometry was used to examine the effects of *Nemania* sp. EL006872 extract, on BaP-induced expression of PD-L1 by H9175 cells. The results showed that increased surface protein level of PD-L1 in BaP exposed condition were diminished by EL006872 extract pretreatment (Figures 1E,F).

### Effect of isolated diterpenes on the transcriptional regulator AhR and the immune checkpoint marker PD-L1 in H1975 cells exposed to BaP

**FIGURE 2:**
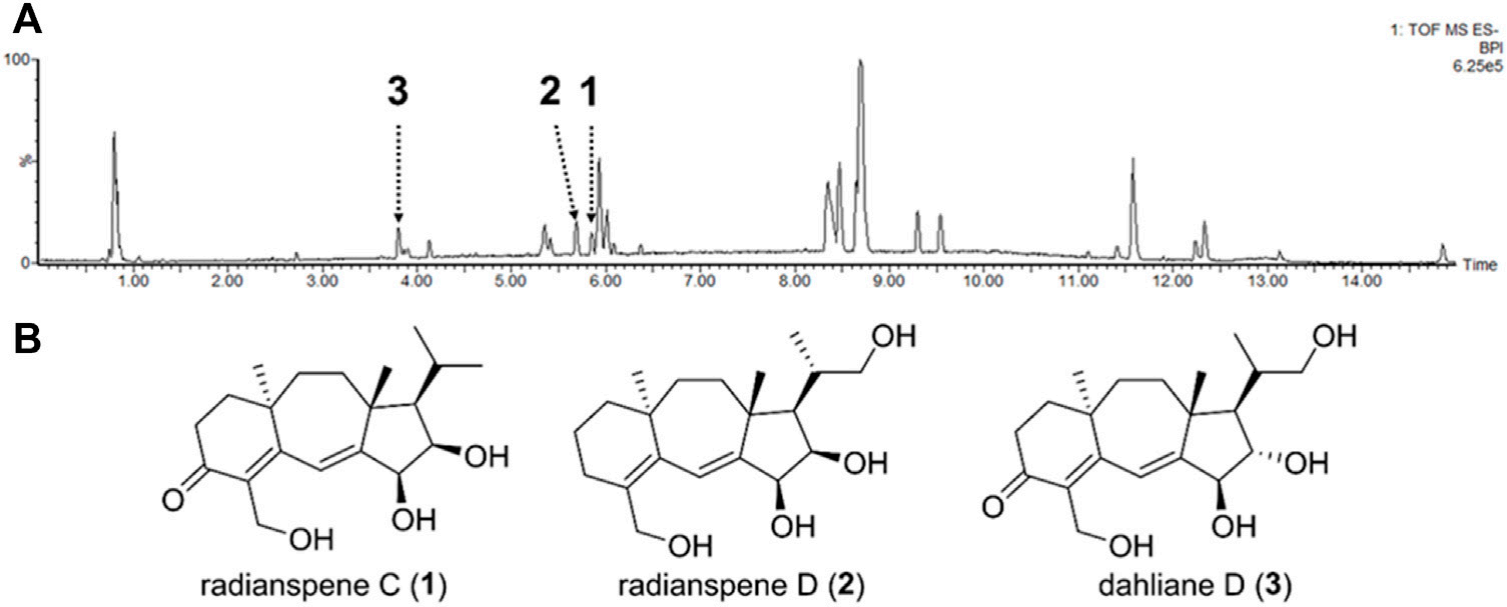
Chemical structures of radianspenes C and D, and dahliane D. **(A)** LC-MS base peak ion chromatogram (negative ion mode) of the *Nemania* sp. EL006872 crude extract. The isolated compounds were denoted. It should be noted that the intensities of chromatographic peaks do not represent absolute quantity of each compound, because the MS signal intensity is heavily affected by ionization efficiency of each molecule. **(B)** Chemical structures of radianspenes C and D, and dahliane D.

LC-MS analysis on the crude extract of *Nemania* sp. EL006872 revealed the presence of multiple compounds (Figure 2A). Successive chromatography yielded three purified compounds, which were identified as radianspenes C and D, and dahliane D (Figure 2B) by comparing their NMR spectra with those reported in the literature (Ou et al., 2012; Wu et al., 2015). Although the LC-MS chromatogram showed other major peaks, they were not able to be purified due to the trace amounts. None of these isolates were cytotoxic to H1975 lung cancer cells up to 30 μM (Supplementary Figure S1); therefore, concentrations of 7.5, 15, and 30 μM (∼2.5, 5, 10 μg/mL) were used in subsequent biological assays.

**FIGURE 3:**
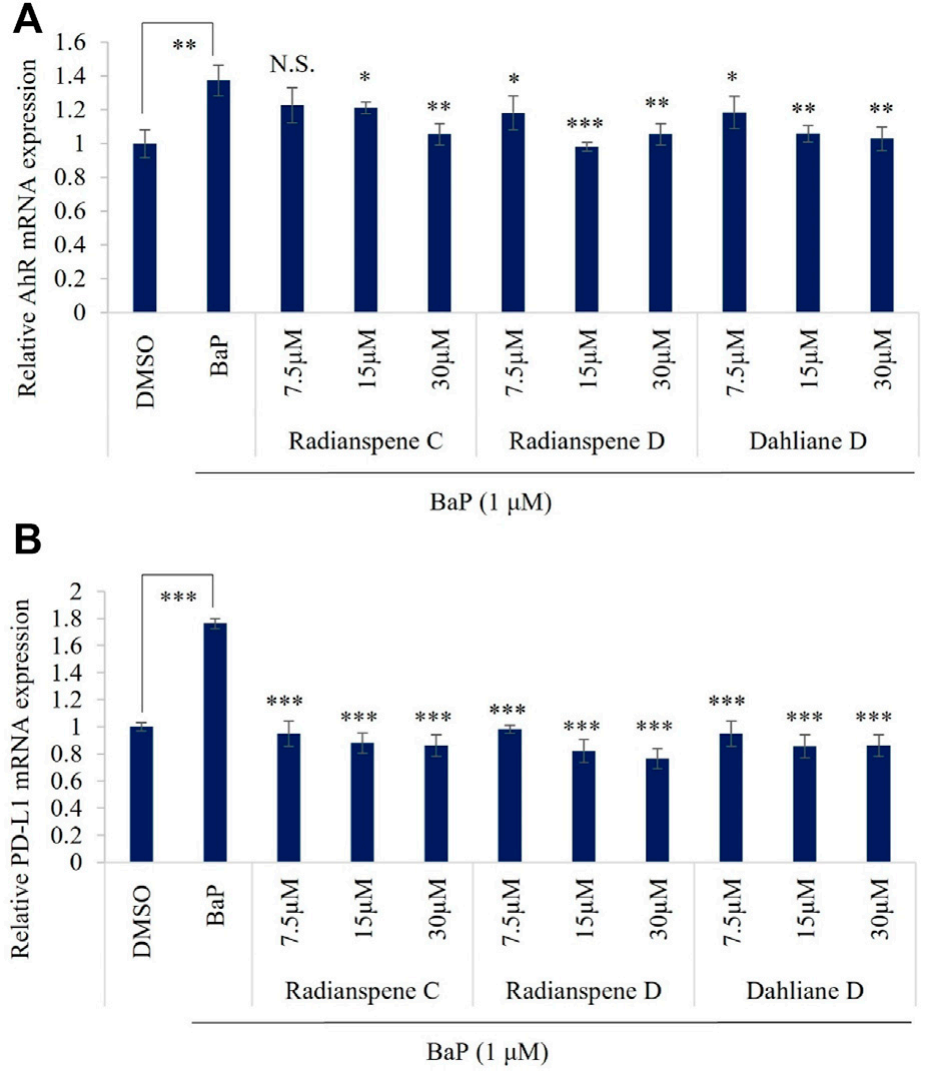
Radianspenes C and D, and dahliane D, suppress benzo[a]pyrene (BaP)-induced expression of AhR and PD-L1 mRNA. H1975 cells were treated with radianspene C and, D and dahliane D, at 7.5, 15, and 30 µM ( ~ 2.5, 5, 10 µg/mL) 4 h prior to exposure benzo[a]pyrene (1 µM) and then cells were incubated for 72 h, AhR **(A)** and PD-L1 **(B)** mRNA level was measured using qRT-PCR. *p < 0.05; **p < 0.01; ***p < 0.001; NS, no significant difference when compared with the BaP-treated group or between indicated group.

**FIGURE 4:**
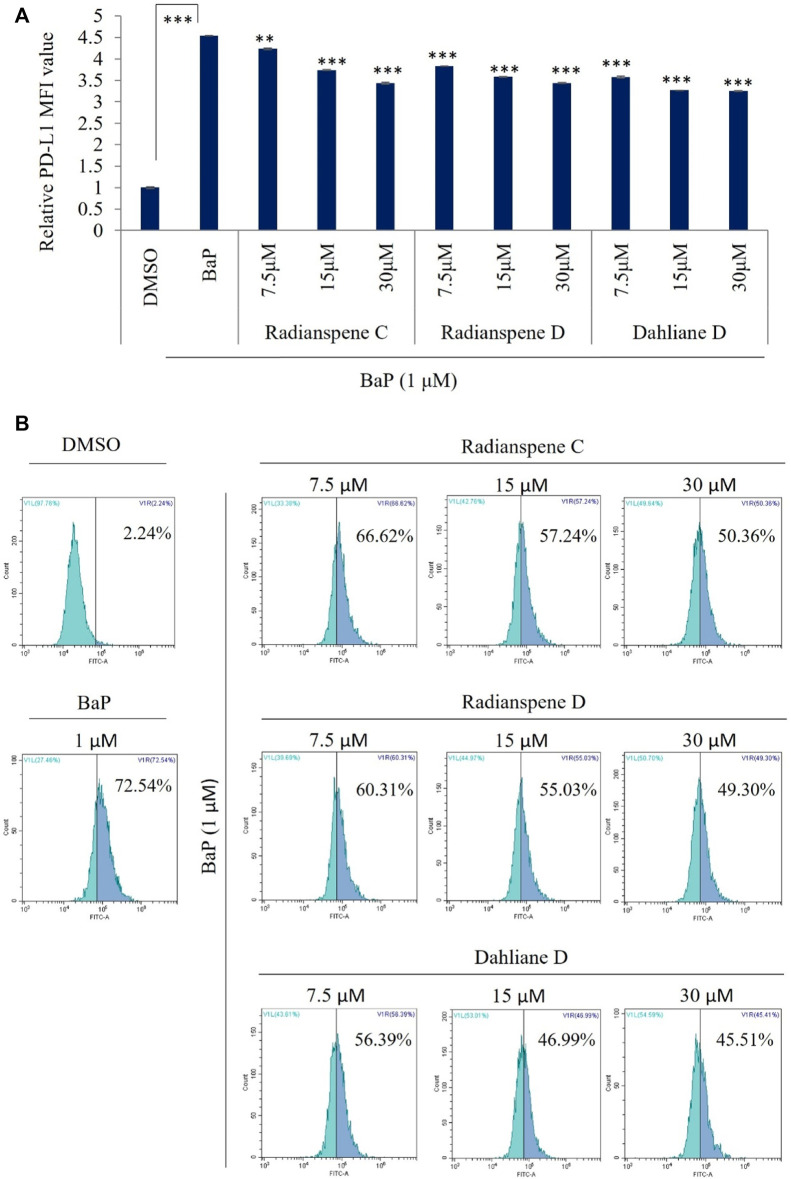
Evaluated the the PD-L1 surface protein activity on benzo[a]pyrene induction condition, treated with radianspenes C and D, and dahliane D. H1975 cells were treated with radianspenes C and, D and dahliane D, at 7.5, 15, and 30 µM ( ~2.5, 5, 10 µg/mL) 4 h prior to exposure benzo[a]pyrene (1 µM) and then cells were incubated for 72 h, and PD-L1 surface protein level was measured using flow cytometry. Relative PD-L1 MFI value was determined by flow cytometry **(A,B)**. MFI: mean fluorescence intensity. *p < 0.05; **p < 0.01; ***p < 0.001; NS, no significant difference when compared with the BaP-treated group or between indicated group.

To evaluate the biological activity of isolated compounds, the expression of AhR and PD-L1 mRNA level were checked as shown in Figures 1C,D. Unexpectedly, our results showed that all of three compounds, radianspenes C and D, and dahliane D, reduced the expression of the AhR and PD-L1 mRNA (Figures 3A,B). To further confirmation and characterization, flow cytometry analysis was performed to investigate the bioactive compounds on cell surface expression of PD-L1 upon exposure of BaP in H1975 cells. The results showed that radianspenes C and D, and dahliane D, downregulated PD-L1 surface protein level in a dose-dependent manner on BaP exposed condition (Figures 4A,B).

### Radianspenes C and D, and dahliane D, reduce expression of multiple immune checkpoint proteins

**FIGURE 5:**
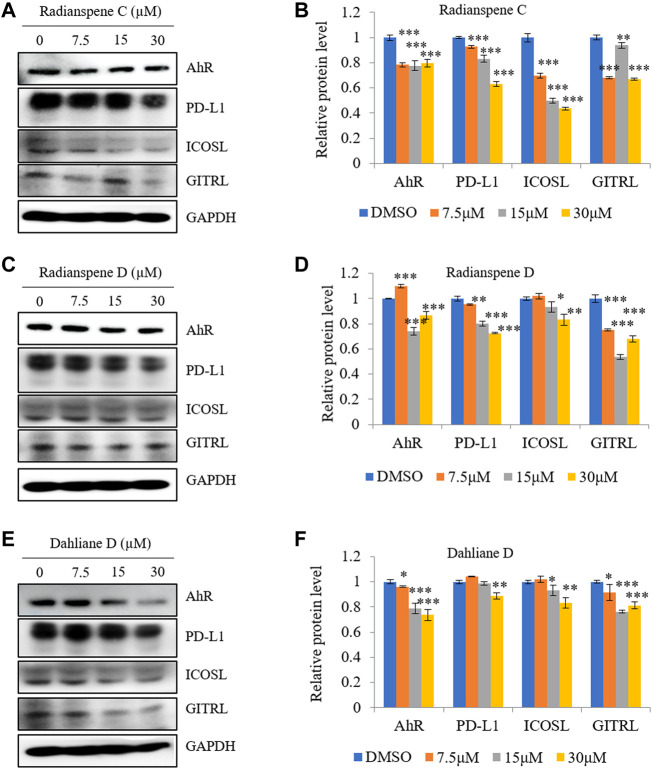
Effect of radianspenes C and D, and dahliane D, on expression of AhR, PD-L1, ICOSL, and GITRL. The level of AhR, PD-L1, ICOSL and GITRL protein by radianspenes C **(A,B)** and D **(C,D)**, and dahliane D **(E,F)** treatment were shown. Data are presented as the mean ± SD, *n* = 3. *p < 0.05; **p < 0.01; ***p < 0.001 compared with the DMSO-treated group.

Next, the inhibitory effects of ELF-derived compounds on expression of multiple immune checkpoint markers (i.e., PD-L1, ICOSL, and GITRL) were evaluated. Expression of PD-L1, GITRL, and ICOSL were downregulated by radianspenes C and D, and dahliane D, in a dose-dependent manner. In addition, the expression of the transcriptional regulator AhR was also investigated. The level of AhR protein decreased upon treat to ELF-derived compounds. Thus, radianspenes C and D, and dahliane D, exert potent inhibitory effects against multiple immune checkpoints (Figure 5).

### Radianspenes C and D, and dahliane D, have anti-proliferative effects in H1975 cells

**FIGURE 6:**
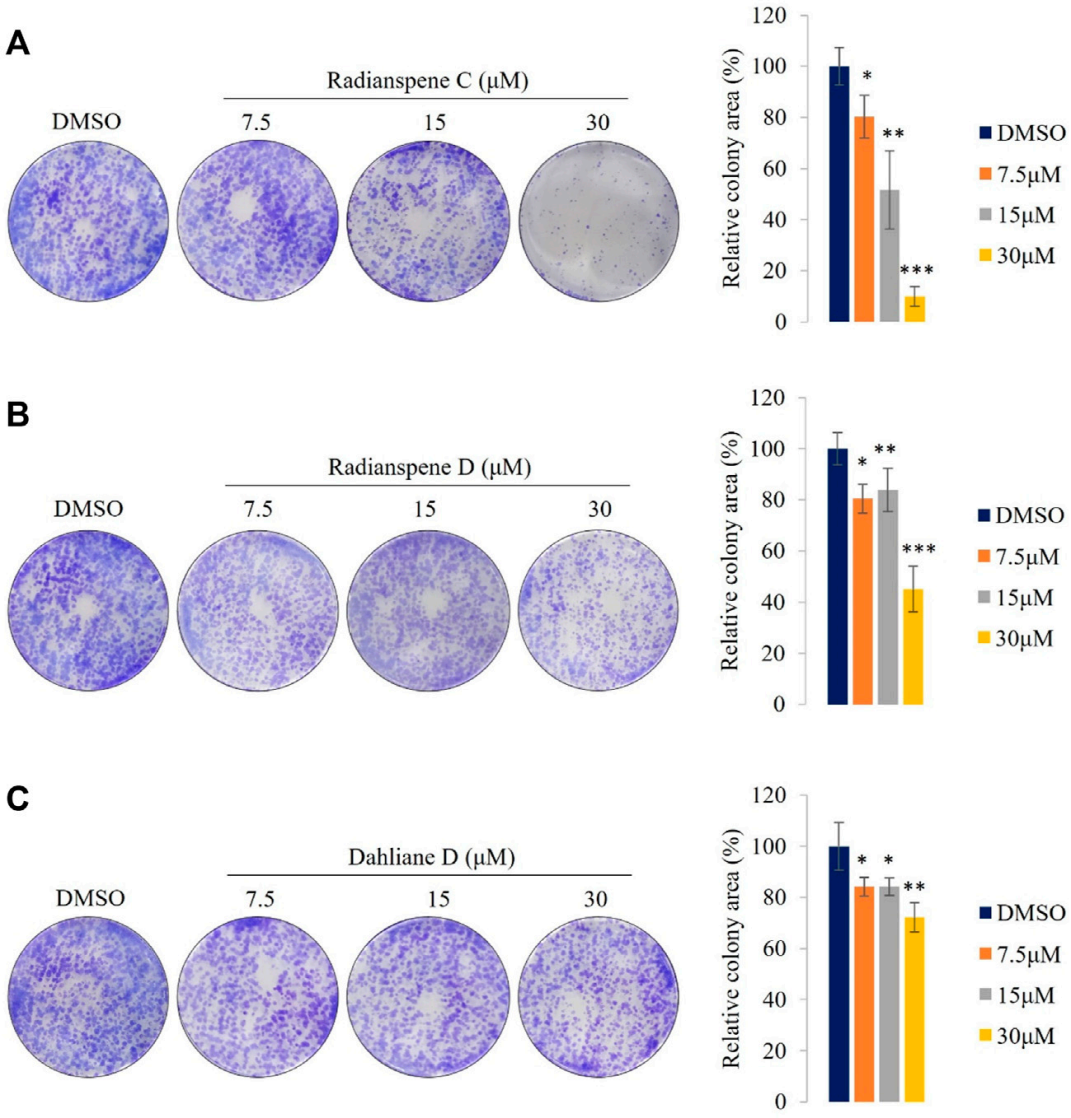
Radianspenes C and D, and dahliane D, show anti-proliferative effects against H1975 cells. **(A)** Radianspene C, **(B)** radianspene D, and **(C)** dahliane D show anti-proliferative activity in a clonogenic assay. Representative images are shown, alongside histograms showing the percentage of colony area in each condition. Data are presented as the mean ± SD, *n* = 3. *p < 0.05; **p < 0.01; ***p < 0.001 compared with the DMSO-treated group.

To investigate the anti-proliferative potential of the ELF-derived compounds *in vitro*, a clonogenic assay was performed in H1975 cells. Cancer cell proliferation and tumor growth are the defining features of cancer progression (Taş et al., 2021). The proliferative capacity of the H1975 cells were suppressed significantly by radianspenes C and D, and dahliane D, in a dose-dependent manner (Figure 6). Thus, the diterpenoid compounds radianspenes C and D, and dahliane D, show anti-proliferative activity. The highest concentration of radianspene C (30 μM) inhibited colony formation to the greatest extent.

## Discussion

**FIGURE 7:**
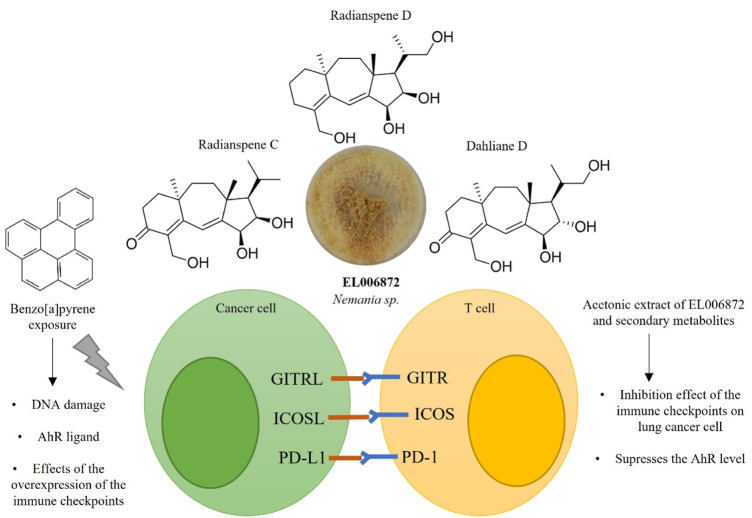
Schematic overview of the immune checkpoint inhibitory activity of diterpenoid compounds isolated from *Nemania* sp. EL006872.

Here, the effects of an ELF extract and isolated secondary metabolites on immune checkpoints in non-small lung cancer cells were investigated. This study identified (Figure 7) 1) the ELF species *Nemania* sp. EL006872 was isolated from *Bryoria fuscescens* (Gyelnik) Brodo and D. Hawksw; 2) *Nemania* sp. EL006872 showed immune checkpoint inhibition activity through suppressing AhR and PD-L1 mRNA and surface protein expression in BaP exposed H1975 cells; 3) radianspenes C and D, and dahliane D were isolated from *Nemania* sp. EL006872 crude extract and found to have bioactivity; 4) radianspenes C and D, and dahliane D suppresses the expression of multiple immune checkpoints including PD-L1, ICOSL, and GITRL; 5) radianspenes C and D, and dahliane D showed anti-proliferative activity.

Radianspenes C and D, and dahliane D, are secondary metabolites (diterpenoids) found in fungi (Zhang and Feng, 2022). A previous study showed that radianspene C from *Coprinus radians* showed cytotoxic effect on MDA-MB-435 human breast cancer cells with an IC_50_ value of 0.91 μM (Ou et al., 2012). Another study isolated the fungus *Verticillium dahliae* from the intestine of the insect *Coridius chinensis*, and identified new guanacastane-type diterpenoids (dahlianes A–K) (Wu et al., 2015; Zhang et al., 2019). Previously, dahlianes A–D were tested against human cancer cell lines (HepG2, MCF-7, and HT-29) and found to be non-cytotoxic, even at a concentration of 50 µM (Wu et al., 2015). Here, it was found that none of the secondary metabolites were toxic effects to H1975 cells at concentrations up to 30 μM (Supplementary Figure S1). The non-cytotoxic effect of radianspenes C, D and dahliane D isolated from the extract on cell viability is consistent with the results of the crude extract. Our results show that the crude extract and pure components can be used safely at a concentration of 10 μg/mL.

Surgery, radiotherapy, and chemotherapy are the standard treatments for cancer; however, their effects are limited, necessitating development of new therapeutic options such as antitumor immunotherapy (Lemjabbar-Alaoui et al., 2015; Jain et al., 2018). Studies carried out under the umbrella of cancer immunotherapy have led to development of strategies that prevent inactivation of T cells by blocking immune checkpoints with an antibody (Pardoll, 2012). In our study, focuses were made on the PD-1/PD-L1, ICOS/ICOSL, and GITR/GITRL pathways in cancer cells. The most studied and well known mechanism is inhibition of PD-L1 on tumor cells, and the PD-1 and CTLA-4 signaling pathways in T cell cells (Kosanić et al., 2013; Borghaei et al., 2015; Horn et al., 2018). CTLA-4 and PD-1 pathway inhibitors include Ipilimumab, Tremelimumab, Pembrolizumab, Nivolumab, Pidilizumab, Durvalumab, and Atezolimab (Buchbinder and Desai, 2016). Previous studies show that PD-L1 is induced by BaP via AhR (Wang et al., 2019). Here, crude extract from *Nemania* sp. EL006872, and isolated secondary metabolites, inhibit expression of PD-L1 and AhR on non-small lung cancer cell exposed to BaP.

The ICOS/ICOSL (CD275/CD278) pathway regulates the Treg population and, subsequently, tumor development (Amatore et al., 2020). Expression of ICOS/ICOSL regulates the CD4^+^, CD8^+^, and FoxP3+ regulatory effector cell populations. Overexpression of ICOS ligand is associated with reduced patient survival (Burmeister et al., 2008; Marinelli et al., 2018). Many studies examined molecules targeting different immune checkpoints. For example, GSK3359609, JTX-2011, MEDI-570, and KY1044 inhibit ICOS (Solinas et al., 2020). Another immune checkpoint marker is GITRL (glucocorticoid-induced tumor necrosis factor receptor-related protein ligand), which plays a role in immune cell activation, survival and signaling (Wang et al., 2021). Targeting the GITRL-GITR pathway activates CD4^+^ and CD8^+^ T cells, thereby promoting tumor suppression (Durham et al., 2017). Studies on the interaction between immune checkpoint signaling pathways and other signaling pathways, and its inhibition in different immunological diseases, are continuing.

## Conclusion

The ELF extract inhibit immune checkpoint ligands in cancer cells exposed to BaP, an environmental pollutant. Radianspenes C and D, and dahliane D, suppress multiple immune checkpoint markers in lung cancer cells. The *Nemania* sp. EL006872 crude extract and its secondary metabolites have the potential for use as support therapies, and to act as templates for development of new target molecules to overcome drug resistance. Our study shows that radianspenes C and D, and dahliane D, can be isolated from ELF and used to conduct research into the treatment of cancer and other diseases related to immune checkpoints. Future studies will examine the molecular mechanisms of action and *in vivo* effects of *Nemania* sp*.* EL006872 secondary metabolites on immunoregulation and tumor growth.

## Data Availability

The original contributions presented in the study are included in the article/Supplementary Material, further inquiries can be directed to the corresponding author.

## Abbreviations

AhR, Aryl hydrocarbon receptor; BaP, benzo[a]pyrene; DMSO, Dimethyl sulfoxide; ELF, endolichenic fungi; GITRL, Glucocorticoid-induced TNFR-related protein ligand; ICOSL, Inducible T Cell Costimulator Ligand; ITS, internal transcribed spacer; MFI, Mean Fluorescence Intensity; PDA, potato dextrose agar; PD-L1, programmed death-ligand 1.
