# Supplementary material for: An acetonic extract and secondary metabolites from the endolichenic fungus Nemania sp. EL006872 exhibit immune checkpoint inhibitory activity in lung cancer cell
Source: Front Pharmacol. 2022 Sep 8;13:986946. doi: 10.3389/fphar.2022.986946 (PMC9495263; doi:10.3389/fphar.2022.986946)
Supplement: Supplementary file 1 [file DataSheet1.docx]

Supplementary Table S1. EL006872 ITS sequence information

| TATGATATGCTTAAGTTCAGCGGGTATTCCTACCTGATCCGAGGTCAACCTATACATTGGGGGGTGTTTTACGGCAGGGCGCCGGGCTTAGGCTACAGGCGATGTGTAAAAGCTACTACGTCTGGAGTGTTAAACCGGCTCCGCCACTGACTTTGGGGAGCTACGGGACAGGGTCCGGTAGGCTCCCAACGCTAAGCAACTGGGGCTTAAGGGTTGAAATGACGCTCGAACAGGCATGCCCACCAGAATACTGGTGGGCGCAATGTGCGTTCAAAGATTCGATGATTCACTGAATTCTGCAATTCACATTACTTATCGCATTTCGCTGCGTTCTTCATCGATGCCAGAACCAAGAGATCCGTTGTTGAAAGTTTTAACTGATTTAGTTATCTTTGTTTCAGAGGTCTAATGCTATACAAAACAGAGTTTCGGGGGCCGTCGGCAGGTTTCGCCTACGAGGGGTAGGGCGGCGCCTGCCGAGGCAACGAGAGGTATGTTCACATGGGTTTGGGAGTTTGTTAGAACTCTATAATGATCCCTCCGCTGGTTCACCAACGGAGACCTTGTTACGA |
| --- |

Table S2: ^1^H NMR (500 MHz) and ^13^C NMR (125 MHz) spectral data of the isolated compounds (CD_3_OD).

|  | Radianspene C | | Radianspene D | | Dahliane D | |
| --- | --- | --- | --- | --- | --- | --- |
| position | δ_C_ | δ_H_ (*J* in Hz) | δ_C_ | δ_H_ (*J* in Hz) | δ_C_ | δ_H_ (*J* in Hz) |
| 1 | 166.7 |  | - |  | 159.2 |  |
| 2 | 120.0 | 6.35 (d, 2.7) | 121.6 | 6.14 (d, 2.2) | 120.2 |  |
| 3 | 163.9 |  | - |  | 166.4 |  |
| 4 | 133.3 |  | - |  | 134.6 |  |
| 5a | 201.2 |  | 28.5 | 2.17 (m) | 201.2 |  |
| 5b |  |  |  | 2.11 (m) |  |  |
| 6a | 34.6 | 2.61 (ddd, 16.5, 11.3, 5.0) | 19.8 | 1.69 (m) | 34.8 | 2.6 (m) |
| 6b |  | 2.41 (ddd, 16.5, 6.6, 4.8) |  | 1.66 (m) |  | 2.43 (m) |
| 7a | 35.8 | 1.87 (m) | 39.2 | 2.18 (m) | 38.9 | 1.94 (m) |
| 7b |  |  |  |  |  | 1.84 (m) |
| 8 | 37.9 |  | - |  | 37.0 |  |
| 9a | 36.9 | 2.41 (m) | 41.8 | 1.23 (m) | 37.4 | 1.48 (m) |
| 9b |  | 1.46 (m) |  | 1.49 (m) |  | 1.46 (m) |
| 10a | 38.8 | 1.93 (m) | 35.8 | 1.71 (m) | 36.0 | 1.84 (m) |
| 10b |  | 1.86 (m) |  | 1.67 (m) |  |  |
| 11 | 45.4 |  | - |  | 46.8 |  |
| 12 | 58.1 | 1.2 (m) | 53.1 | 1.39 (m) | 53.4 | 1.48 (m) |
| 13 | 74.7 | 4.08 (dd, 4.4, 3.6) | 74.2 | 4.08 (dd, 4.4, 3.5) | 74.2 | 4.13 (m) |
| 14 | 76.3 | 4.42 (dd, 4.4, 2.7) |  | 4.36 (m) | 76.4 | 4.43 (dd, 4.4, 2.7) |
| 15a | 56.2 | 4.29 (d, 11.5) | 63.5 | 4.22 (d, 12.3) | 56.2 | 4.29 (d, 11.5) |
| 15b |  | 4.25 (d, 11.5) |  | 3.88 (d,12.3) |  | 4.27 (d, 11.5) |
| 16 | 26.4 | 1.21 (s) | 26.5 | 1.01 (s) | 26.4 | 1.21 (s) |
| 17 | 20.2 | 1.18 (s) | 18.8 | 1.18 (s) | 20.4 | 1.19 (s) |
| 18 | 26.6 | 2.06 (m) | 33 | 2.06 (m) | 33.2 | 2.05 (m) |
| 19a | 23.4 | 1.06 (d, 6.6) | 67.6 | 3.69 (dd, 11.1, 4.3) | 67.83 | 3.68 (dd, 10.6, 3.8) |
| 19b |  |  |  | 3.61 (dd, 10.7, 5.1) |  | 3.64 (dd, 10.6, 5.1) |
| 20 | 23.5 | 1.00 (d, 6.6) | 18.1 | 1.06 (d, 6.8) | 18.3 | 1.07 (d, 6.8) |


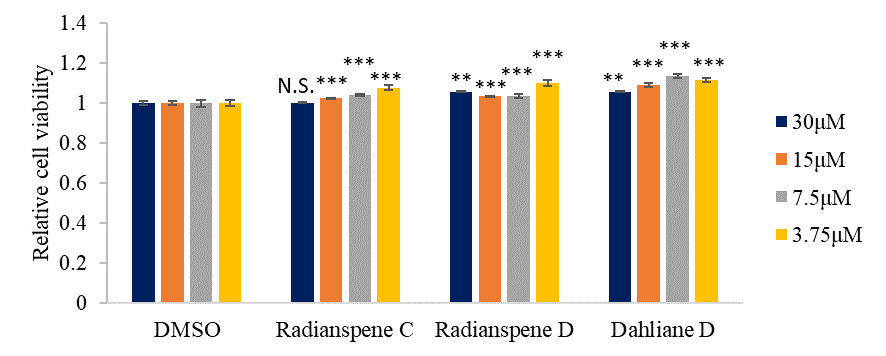


Figure S1. Effect of Radianspene C-D and Dahliane D on the cell viability on H1975 lung cancer cell line.

Cell viability with dose-dependent Radianspene C-D and Dahliane D. Cell viability was measured using an MTT (3-(4,5-Dimethylthiazol-2-yl)-2,5-Diphenyltetrazolium Bromide) assay. Data are presented as mean ± SD, n = 3. *p < 0.05; **p < 0.01; ***p < 0.001.


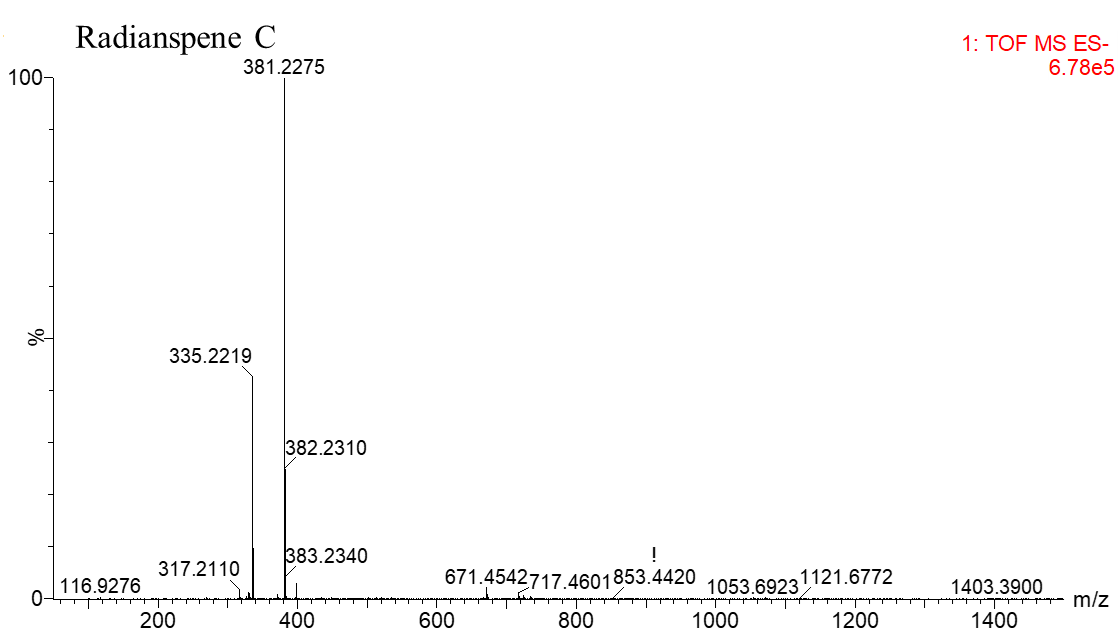


Figure S2. HRESIMS spectrum of radianspene C


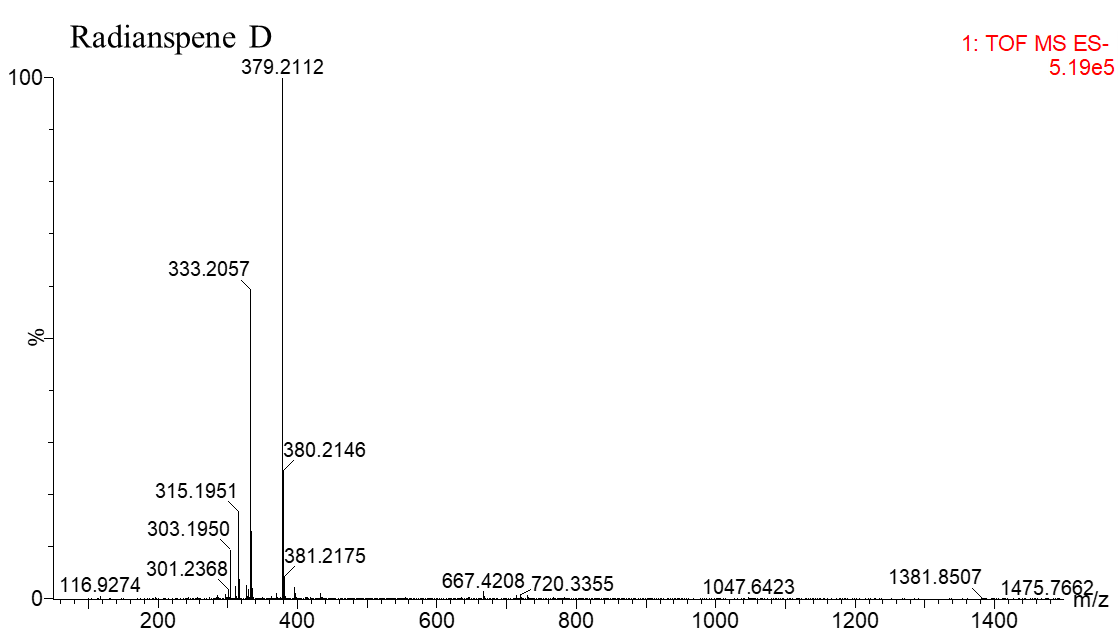


Figure S3. HRESIMS spectrum of radianspene D


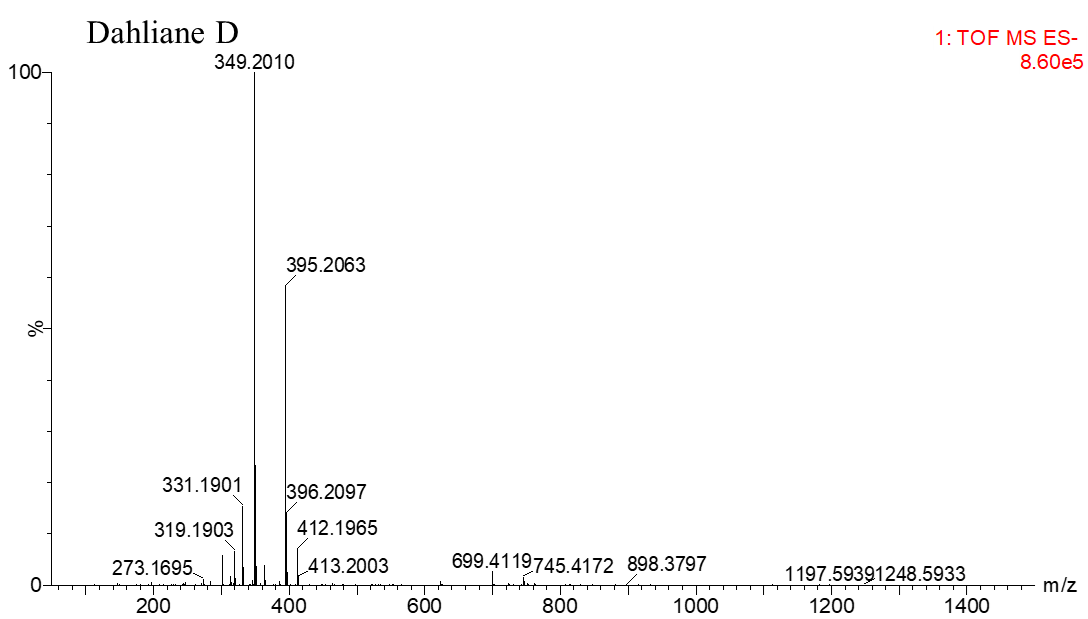


Figure S4. HRESIMS spectrum of dahliane D


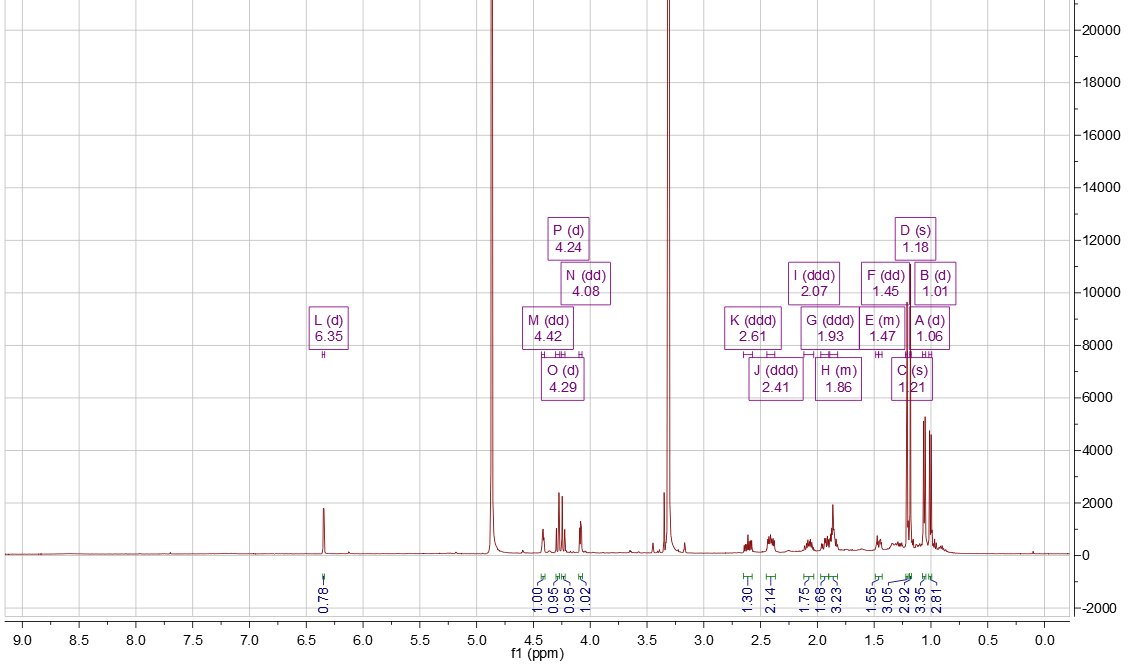


Figure S5. ^1^H NMR spectrum of radianspene C


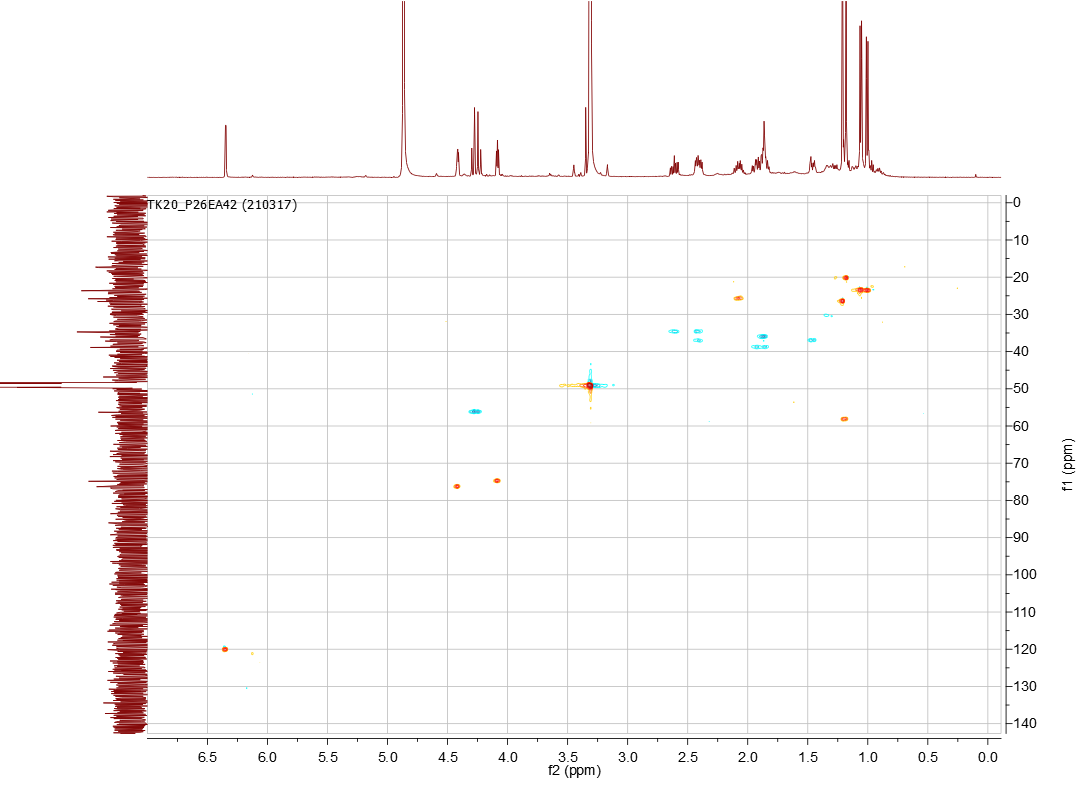


Figure S6. HSQC spectrum of radianspene C


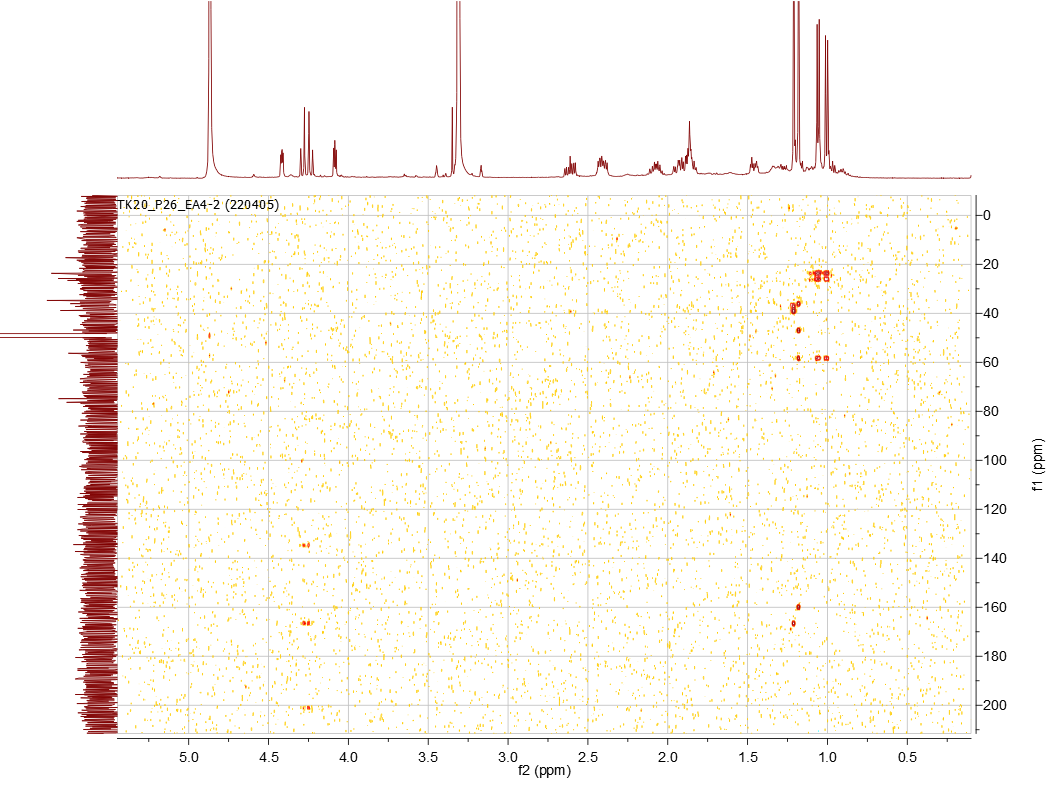


Figure S7. HMBC spectrum of radianspene C


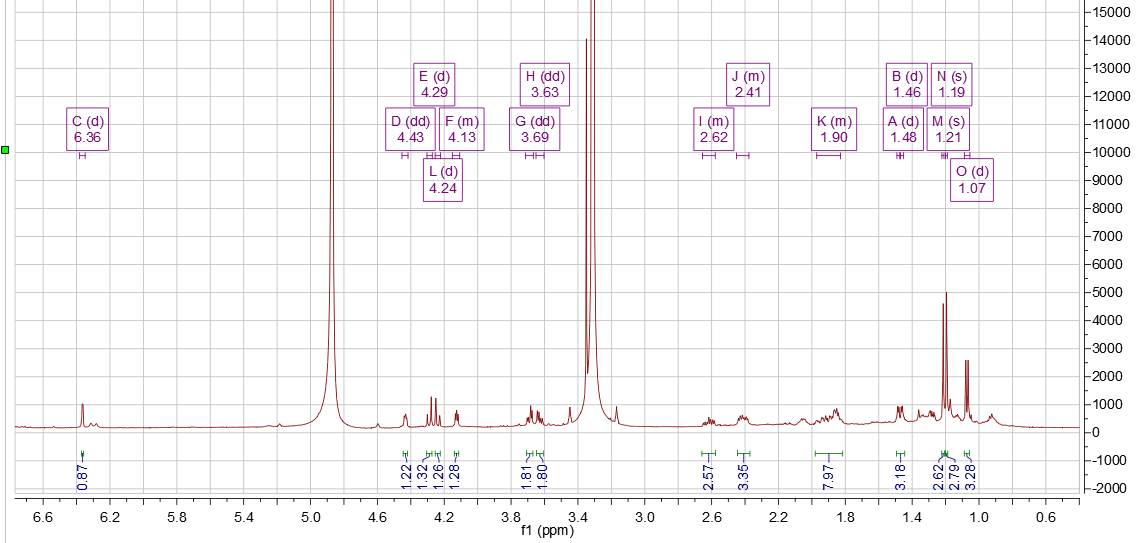


Figure S8: ^1^H NMR spectrum of Dahliane D


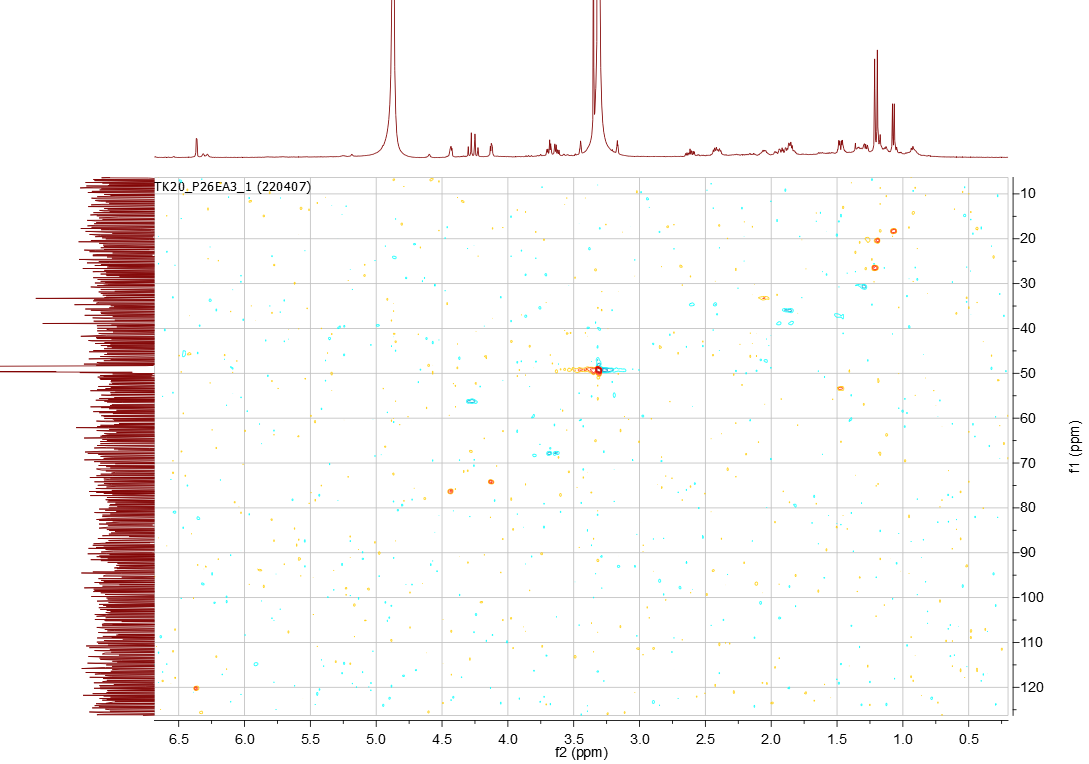


Figure S9. HSQC spectrum of Dahliane D


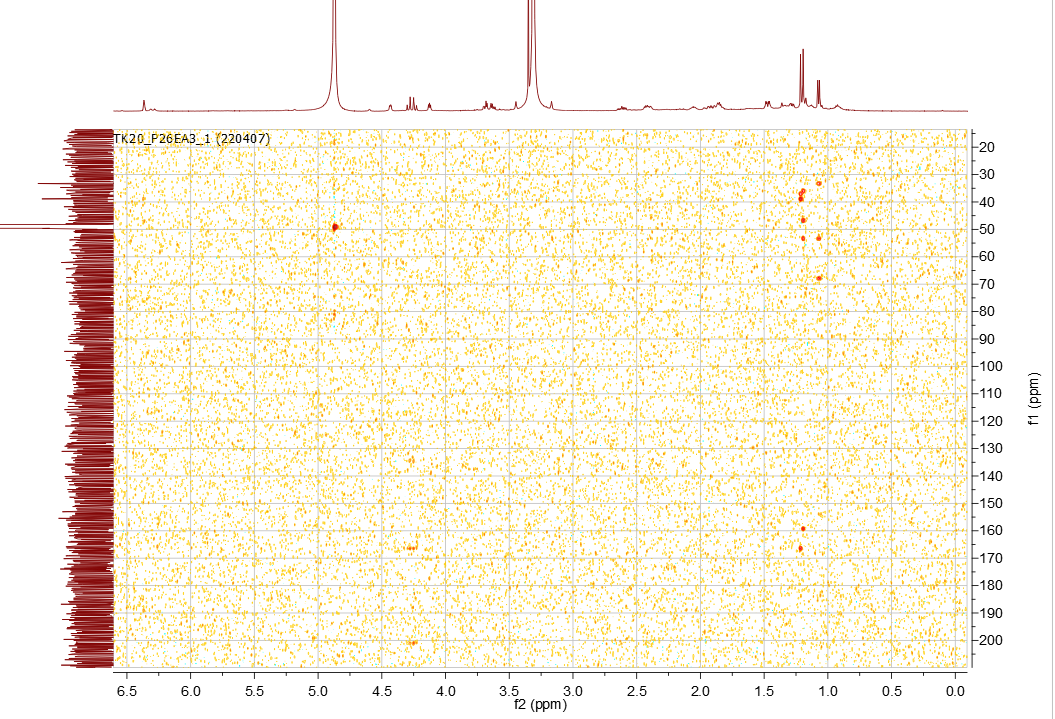


Figure S10. HMBC spectrum of Dahliane D


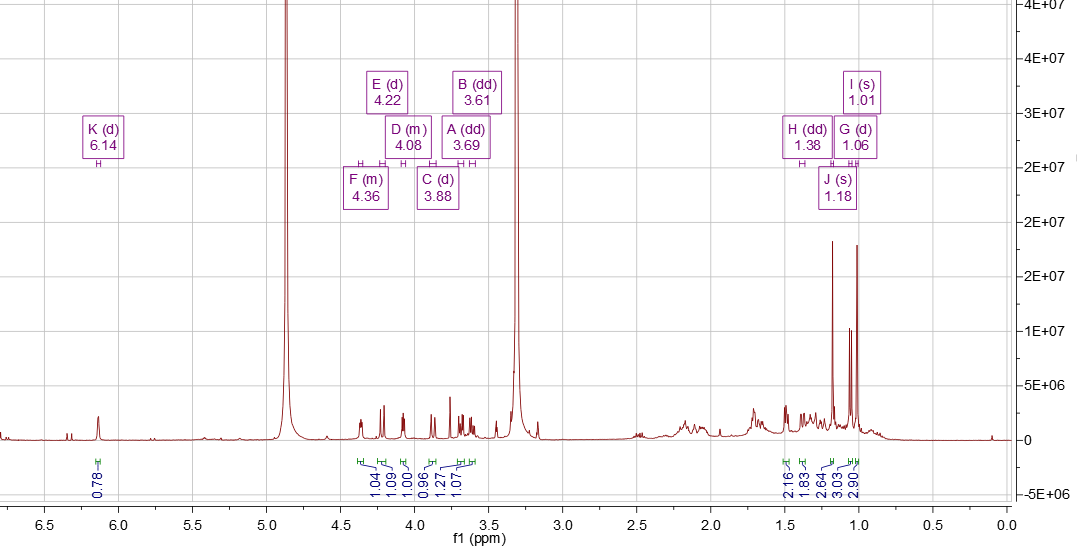


Figure S11. ^1^H NMR spectrum of radianspene D


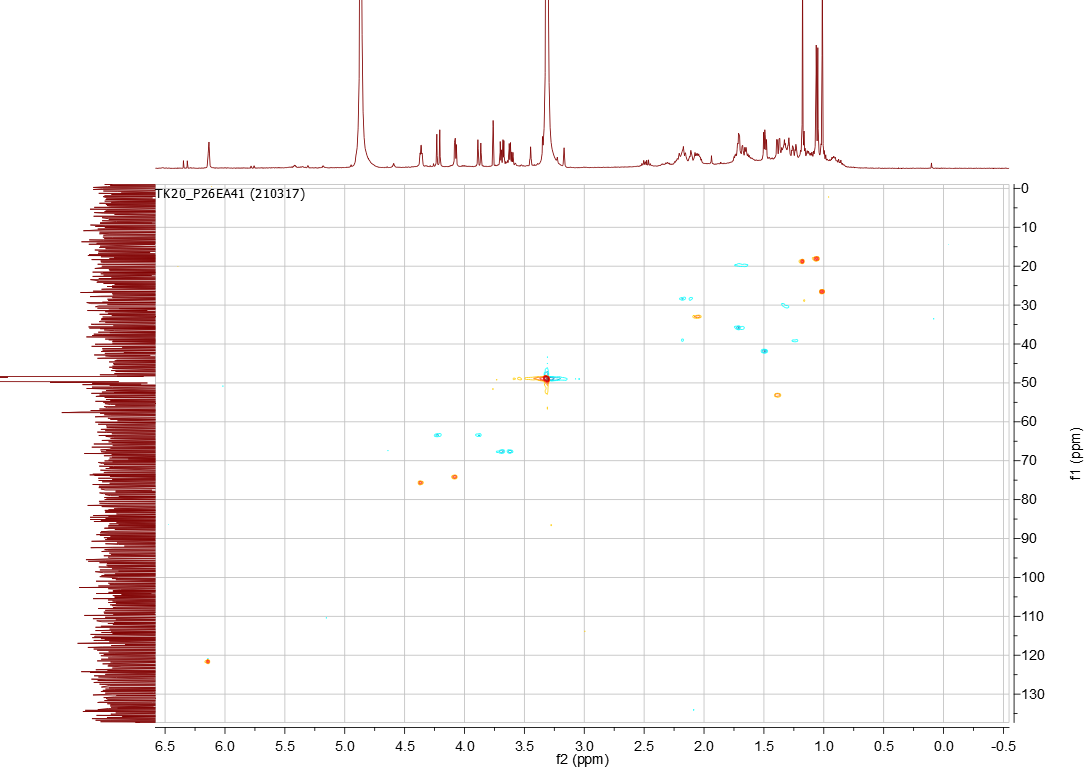


Figure S13. HSQC spectrum of radianspene D
